# Compensation of overall physical activity in (pre)adolescent girls – the CReActivity project

**DOI:** 10.1186/s13690-022-01002-1

**Published:** 2022-12-02

**Authors:** Franziska Beck, Ulrich Dettweiler, David Joseph Sturm, Yolanda Demetriou, Anne Kerstin Reimers

**Affiliations:** 1 Department of Sport Science and Sport, Friedrich-Alexander-Universität Erlangen-Nürnberg, Gebbertstr. 123B, 91058 Erlangen, Germany; 2 Cognitive and Behavioral Neuroscience Lab, University of Stavanger, 4036 Stavanger, Norway; 3 Department of Sport and Health Sciences, Technical University of Munich, Georg-Brauchle-Ring 60, 80992 Munich, Germany

**Keywords:** Compensation, Girls, (pre)adolescents, Negative, Positive, Physical activity

## Abstract

**Background:**

According to the ActivityStat hypothesis more physical activity (PA) in one timespan is compensated by increased sedentary time (ST) in the following timespan and vice versa to maintain an overall stable PA level. Until now, existing literature revealed inconsistent results regarding compensatory behaviour across children and adolescents. Thus, the aim of the present study is (1) to investigate whether ST in the morning is compensated by active behaviour in the afternoon and (2) whether ST during the week is compensated by active behaviour during the weekend in (pre)adolescent girls. Additionally, we aimed to differentiate between positive and negative compensatory behaviour and examine whether it is moderated by socioeconomic status (SES), age or weight status.

**Methods:**

The participants were 370 sixth grade school girls (mean age 11.6 years) from Munich that participated in the CReActivity study, a school based intervention study aiming to identify the mechanisms of behavioural changes in PA among girls. ST and PA were measured over seven consecutive days using accelerometery. Descriptive determination of compensatory behaviour, as well as Bayesian multivariate multilevel analysis were conducted with data clustered on the individual (ID), class and school level.

**Results:**

Descriptive analysis revealed rather constant compensatory behaviour of about 60% for after-school days and weekends over all observation points. However, regarding all girls, compensation was predominantly negative. Differentiated analysis indicated that all girls with low ST levels in the morning or on weekdays, compensated for this behaviour with lower PA levels in the afternoon or on weekends. Multilevel covariate analysis indicated great variability between the participants. Furthermore, differences in compensatory behaviour can also be seen on class and school levels. Interestingly, PA compensatory behaviour is not associated with age, weight status or SES.

**Conclusion:**

Our findings could neither confirm nor reject the ActivityStat Hypothesis. Overall, due to the great variability across the girls, it seems that compensation depends on individual factors. In the future, to prevent negative compensation, school-based interventions that have the potential to provide opportunities to be physically active, should not neglect (pre)adolescents’ leisure time behaviour.

**Trial Registration:**

DRKS00015723 (date of registration: 2018/10/22 retrospectively registered).

## Background

Physical activity (PA) is described as “any bodily movement produced by skeletal muscles that result in energy expenditure” [1] and is an important source for overall health in children and adolescents [2]. The long-term effects of PA are well documented and indicate an increase in physical fitness as well as improved cardiovascular [3] and cardio metabolic [2, 3] health. Besides the physiological health benefits, PA is associated with better mental health [4, 5]. In order to support children and adolescents in achieving these benefits, the World Health Organization (WHO) developed PA recommendations for children and adolescents, stating a desired average of 60 min/day of moderate-to-vigorous intensity, mostly aerobic PA, across the week [6]. Furthermore, vigorous-intensity aerobic activities, as well as those that strengthen muscle and bone, should be incorporated at least 3 days a week [7]. Nevertheless, PA levels of children and adolescents in many regions worldwide do not meet the WHO recommendations [8–10]. Specifically, with increasing age, the prevalence of guideline compliance seems to decrease [11–13], and the difference between boys and girls consistently widens from childhood to adolescence [14]. Therefore, (pre)adolescent girls reveal lower PA levels compared to boys at this age [9, 15–18] and represent a high-risk population related to PA [19].

In addition to age and gender, further sociodemographic variables must be taken into account to understand PA levels in children and adolescents. In particular, regarding the socioeconomic status (SES) of the parents, including available resources and family lifestyle, various studies have shown that children in all age groups from families with a higher SES are more physically active compared to children from families with a lower SES [20–22]. Results from the German MoMo-Study confirmed these results with regards to total PA and organised PA. Nevertheless, in settings such as unorganised sports or extracurricular sports an inverse relationship was found [23]. In addition to the influence of SES and age, (pre)adolescents with higher weight status are less active than children with lower weight status [12,24].

Numerous efforts to promote PA among children exist across different settings, including schools, family environment or local community settings, but efficacy in the short and long term has widely varied [25, 26]. Additionally, the WHO stated that global efforts seem to miss the goal and that further action is needed [10].

One reason for the limited effects of current efforts could be the mechanisms of compensation as postulated in the ActivityStat hypothesis [27]. The assumptions of this hypothesis are generally not taken into account when promoting PA in children and adolescents. It focuses on the potential effect of the intrinsic biological control that underpins PA and energy expenditure (EE): an imposed increase or decrease in PA in one domain/timespan might induce a compensatory change in the opposite direction, i.e., in another domain/timespan, in order to maintain a stable level of PA or EE over the time [27]. Compensation can occur by increasing or decreasing the intensity, frequency and/or duration of time spent in PA or inactivity [28]. Compensation can occur as positive or negative. Positive compensation is defined as an increase in PA levels in one time span following low PA levels in another time span. In particular, Tudor-Locke, Lee [29] indicated higher PA levels after school in girls that had low PA levels in school. Negative compensation is defined as a decrease in PA levels following high PA levels. This can often be seen for instance after interventions in which PA levels are increased in one timespan (e.g., in interventions promoting PA at school) [30–37].

Gomersall, Rowlands [27] were the first to publish a review about compensatory behaviour in children, adolescents and adults. The authors provided an overview of experimental studies investigating compensation with objective and subjective measurement methods, but the results were inconclusive. On this basis, our own review of compensation or displacement in children and adolescents [38] updated the existing overview by including diverse study designs with solely objective measurements and the focus on children and adolescents. Additionally, in our review we differentiated between various subgroups and summarised existing literature related to compensatory behaviour in children and adolescents across different contexts (physical education (PE), interventions, daily PA, organised sports club participation, active travel) [38]. Our data synthesis revealed that half of the studies support and half of the studies refute compensation of PA. A review investigating compensation in youths and adults revealed inconsistent results, but with one third of youths showing compensatory behaviour [39]. Daily PA includes PA levels across a regular school day without any PE, interventions or sports club participation. In this context, half of the studies indicated a negative compensatory behaviour [40–46]. Furthermore, based on sociodemographic variables, potential moderators of compensation were investigated in only a few included studies. To date, studies focused on different age groups [41, 47, 48] and gender [29, 47–51]. Compensatory behaviour was indicated across all age spans from preschool children [52] to adolescents [48, 53] as well as in boys and girls, and there are even a few studies supporting compensation only in girls and not in boys [29, 49, 50, 54]. Related to weight status, studies revealed no differences in compensatory behaviour across normal and overweight children and adolescents [33, 36, 55–57]. To the best of our knowledge, other moderators such as SES have not been yet investigated within a context of compensatory behaviour.

A large body of scientific literature on the compensatory behaviour of PA is available but shows inconclusive results. Furthermore, previous research has focused sparsely on differences in compensatory behaviour related to sociodemographic variables. The aim of the present study is thus to investigate, in (pre)adolescent girls, (1) whether sedentary time (ST) during the morning is compensated by active behaviour in the afternoon and (2) whether ST during the week is compensated by active behaviour during the weekend. Additionally, we aim to examine whether compensatory behaviour is moderated by SES, age and/or weight status.

## Methods

### Study design

The present study is a secondary data analysis of baseline data from the CReActivity project, a school based intervention study aiming to identify the mechanisms of behavioural changes in PA among girls through a self-determination theory-based intervention in PE [58]. The study was approved by the Ethics Committee of the Technical University of Munich (protocol code 155 / 16S) and was in accordance with the 1964 Declaration of Helsinki. All participants as well as one of their parents provided written informed consent for study participation.

### Participants

All secondary schools (Realschulen) in and around Munich were contacted one year before the CReActivity study was implemented. Inclusion criteria for the study were secondary schools and female-only sixth-graders who participated actively in mono-educated PE. Exclusion criteria only existed on a school level and included PE lesson for boys, no gym as well as insufficient or unqualified teachers. Principals and parents’ councils from 15 schools provided informed consent. The researchers gave the letter asking for consent to participate in the data collection process to the PE teachers, who distributed them to the children. The children then forwarded the letters to their parents, as both children and their parents had to provide written informed consent. Five hundred and seven sixth-grade female students from 32 single-sex/gender PE classes were enrolled in this study. Valid baseline data was collected from 370 girls aged 9–14 years.

### Data collection

Data collection took place between October and December in the school years 2017/2018 and 2018/2019, respectively. The questionnaire data was collected during PE lessons held in the gym. After providing instructions, a member of the research team distributed the accelerometers to all eligible students and supervised the completion of the paper–pencil questionnaire. After that a research assistant assessed the anthropometric data using a weight scale and stadiometer. The girls were asked to wear an accelerometer for 7 consecutive days. On week- and weekend days, girls were advised to put the devices on in the morning after getting out of bed and wear them until 9 p.m. or just before bedtime, except during water-based activities. One week later, the PE teacher collected the devices from the students and forwarded them to the research team.

### Measurements

#### Socioeconomic status

Participants reported parents’ occupations and job descriptions. The answers were classified by referring to the International Socioeconomic Index of occupational status (ISEI), which is based on the International Standard Classification of Occupation 2008 (ISCO-08) [59]. When the jobs of both parents could be classified, the job with the higher ISEI was considered (HISEI). Vague answers made a definite classification impossible, reducing the number of HISEI values. Written answers of the parental SES were coded by a trained committee of four student research assistants according to the International Standard Classification of Occupation 2008 (ISCO‐08). After revision by two researchers, open conflicts were solved and coded under consideration of the International Socio‐Economic Index (ISEI) score [59].

#### Age and weight status

Based on the self-reported birth date participants’ age on the first school day of the respective school year was calculated in years. Stature was measured to the nearest 0.1 cm and body weight was measured to the nearest 0.1 kg. Participants were measured wearing sports clothes but without shoes. Body mass index (BMI, kg*m^−2^) was calculated as an indicator of weight status.

#### Physical activity and sedentary time measurement

PA and ST were measured with accelerometers (ActiGraph models GT3X – wGT3X-BT; Pensacola, FL, USA). The devices were worn on the right hip with an elastic belt. The sampling rate was set to 30 Hz. Further details have previously been described in detail [60].

#### Physical activity-related environment

Environmental factors of relevance for PA were assessed with ten items of the ‘Assessing levels of Physical Activity and Fitness at Population level’ (ALPHA) short scale [61]. The scale has been translated and was pilot tested with sixth grade students in Germany. In the validation study the ALPHA short exhibited a stable test–retest reliability with an intra-class correlation coefficient (ICC) of 0.73 [61]. The questionnaire includes ten statements related to the neighbourhood built environment. This means the area around participants’ home that can be reached on foot within a maximum of 15 min. Girls were able to answer yes, no or don’t know. The higher the value, the more places can be reached by foot within the walkable neighbourhood (15 min).

### Accelerometer data reduction

ActiGraph data were downloaded and processed into 1 s epochs using ActiLife. The algorithm by Choi, Liu [62] classifies 90 consecutive minutes of zero counts per minute as non-wearing bouts, allowing for up to 2 min of less than 100 counts per minute (cpm) (within 30 min). Participants’ PA data were regarded as valid if at least 8 h of wear time were recorded on at least three weekdays and one weekend day. Weekends contained data from 7 a.m. to 9 p.m., while weekdays were separated into morning from 7 a.m. to 1 p.m. and afternoon from 1 p.m. till 9 p.m.. According to the cut-points by Hänggi, Phillips [63], we estimated the daily average ST (< 180 cpm), light PA (LPA) (181–3360 cpm) and moderate-to-vigorous PA (MVPA) (> 3360 cpm) for each participant. For the statistical analyses, relative wear time, i.e. proportions of ST, LPA, and MVPA, was calculated.

### Statistical analysis

#### Analytic strategy

The determination of compensatory physical activity behaviour (CPB) is first performed with the descriptive data operationalising formulas for different types of compensation. In a second step, we fit inferential models to analyse the impact of covariates on the physical activity levels in the compensatory time periods.

#### Descriptive statistics

Descriptive statistics were calculated for all study variables, with means (*M*) and standard deviation (*SD*) for continuous variables. For afternoon vs. morning analysis, the 370 girls produced wear time for seven days with 1,146 valid observation points, whereas weekday and weekend analyses were conducted with one value from each girl. In this context, observation points refer to the fact that the girls can produce values on several days in the morning and in the afternoon over a period of seven days.

#### Determination of CPB

CPB is directly derived from the relative PA and ST data and can be defined in terms of positive and negative compensations. In this context, compensation is positively negotiated when higher ST levels in one time-period are followed by higher PA levels in another. On the other hand, a negative compensation describes lower ST in one time-period being compensated by lower PA in the other. CPB can be applied to afternoon (after school) PA/ST behaviour with respect to morning behaviour, and weekend behaviour with respect to week-day behaviour. We defined CPB to be displayed by the participants if the respective physical behaviour pattern in one time-period was at least 1.645 standard deviations different (90% CI) from the other pattern in the other time-period. This relationship is mathematically expressed in the following equations:

$$\mathrm{Positive}\;\mathrm{compensation}:\;\mathrm{if}\;{\mathrm{ST}}_{\mathrm{afternoon}}<{\mathrm{ST}}_{\mathrm{morning}},\;\mathrm{then}\;\left({\mathrm{LPA}}_{\mathrm{afternoon}}+{\mathrm{MVPA}}_{\mathrm{afternoon}}\right)-\left({\mathrm{LPA}}_{\mathrm{morning}}+{\mathrm{MVPA}}_{\mathrm{morning}}\right)>1.645\times\mathrm{sd}({\mathrm{LPA}}_{\mathrm{morning}}+{\mathrm{MVPA}}_{\mathrm{morning}})$$

$$\mathrm{Negative}\;\mathrm{compensation}:\;\mathrm{if}\;{\mathrm{ST}}_{\mathrm{afternoon}}>{\mathrm{ST}}_{\mathrm{morning}},\;\mathrm{then}\;\left({\mathrm{LPA}}_{\mathrm{afternoon}}+{\mathrm{MVPA}}_{\mathrm{afternoon}}\right)-\left({\mathrm{LPA}}_{\mathrm{morning}}+{\mathrm{MVPA}}_{\mathrm{morning}}\right)<1.645\times\mathrm{sd}\left({\mathrm{LPA}}_{\mathrm{morning}}+{\mathrm{MVPA}}_{\mathrm{morning}}\right)$$

$$\mathrm{Positive}\;\mathrm{compensation}:\;\mathrm{if}\;{\mathrm{ST}}_{\mathrm{weekend}}<{\mathrm{ST}}_{\mathrm{weekday}},\;\mathrm{then}\;\left({\mathrm{LPA}}_{\mathrm{weekend}}+{\mathrm{MVPA}}_{\mathrm{weekend}}\right)-\left({\mathrm{LPA}}_{\mathrm{weekday}}+{\mathrm{MVPA}}_{\mathrm{weekday}}\right)>1.645\times\mathrm{sd}\left({\mathrm{LPA}}_{\mathrm{weekday}}+{\mathrm{MVPA}}_{\mathrm{weekday}}\right)$$

$$\mathrm{Negative}\;\mathrm{compensation}:\mathrm{if}\;{\mathrm{ST}}_{\mathrm{weekend}}>{\mathrm{ST}}_{\mathrm{weekday}},\;\mathrm{then}\;\left({\mathrm{LPA}}_{\mathrm{weekend}}+{\mathrm{MVPA}}_{\mathrm{weekend}}\right)-\left({\mathrm{LPA}}_{\mathrm{weekday}}+{\mathrm{MVPA}}_{\mathrm{weekday}}\right)<1.645\times\mathrm{sd}\left({\mathrm{LPA}}_{\mathrm{weekday}}+{\mathrm{MVPA}}_{\mathrm{weekday}}\right)$$

**Fig. 1:**
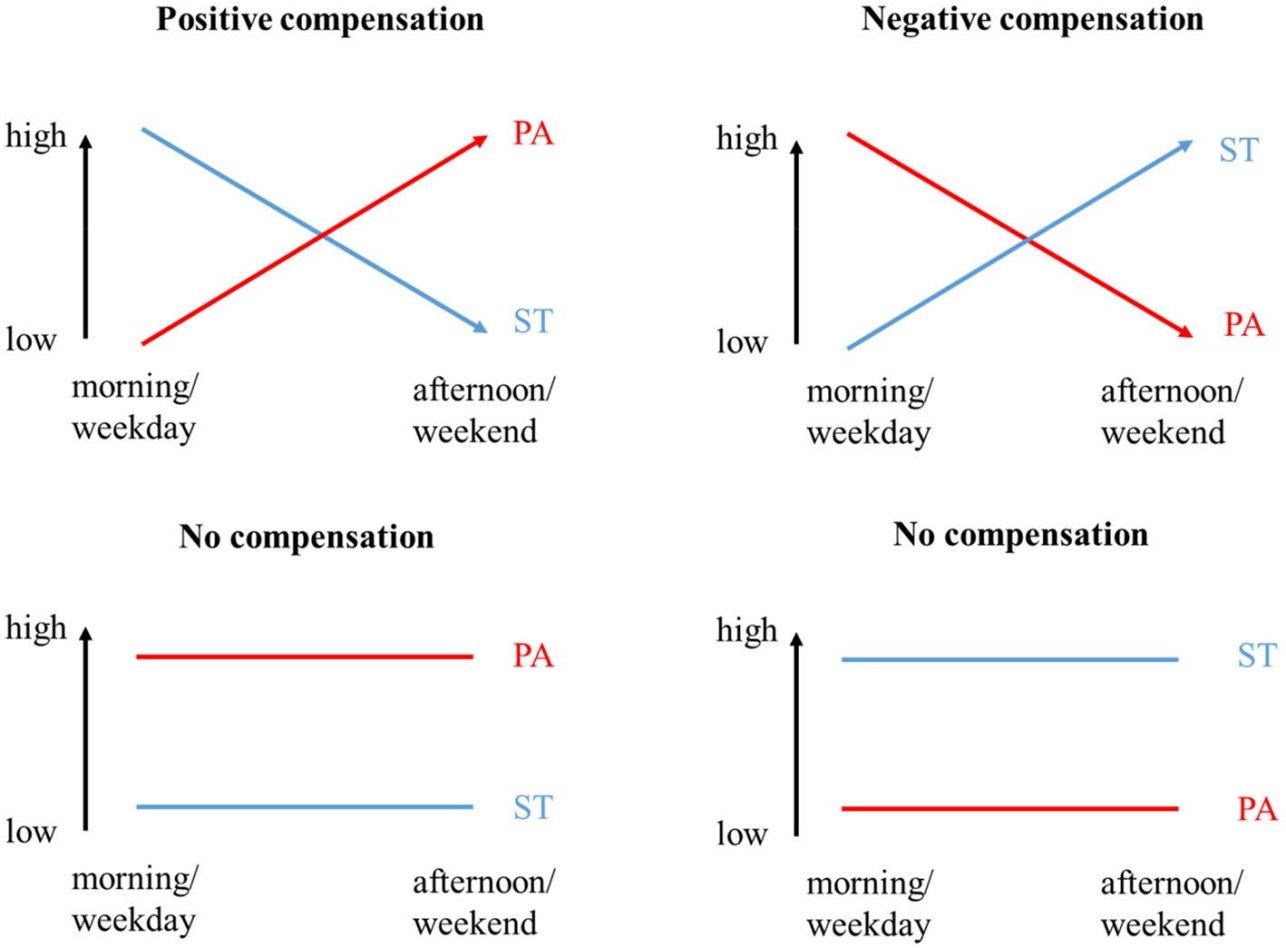
Illustration of positive, negative and no compensation throughout the whole day/week

To better understand compensatory behaviour, the equations for the determinations of CPB are illustrated in the following figures. In addition to the compensatory behaviour, we illustrate what non-compensatory behaviour looks like (Fig. 1).

#### Covariate analysis

To investigate the association between relative ST in the morning and relative PA behaviour in the afternoon (for weekend analysis: relative ST during the week and relative PA behaviour on the weekends) with respect to weight status, age and SES, we fitted a series of multivariate multilevel models. These models were controlled for the built environment factor. We chose a Bayesian approach as it gives more flexibility in the model specification and outperforms maximum likelihood estimation [64]. To ease model convergence and the interpretation of the results, all continuous variables have been normalised (z-standardised). Thus, the scale-free parameter estimates can also be interpreted as effect-sizes.

Since the data are clustered into multiple levels, i.e. observations nested in individuals, who are in turn nested in classes and schools, varying intercepts have been calculated for each level. ICC(1) determines the amount of variance that can be explained by simply clustering the data on the respective level. If the correlation is near zero, the observations within clusters are no more similar than observations from different clusters, hence clustering is not necessary. A threshold of ICC(1) > 0.05 is used to determine whether or not the cluster-level should be accounted for (Table 1).

**Table 1:** ICC(1) for LPA and MVPA in the afternoon and on the weekend across different clusters

	**LPA**			**MVPA**		
	*ID*	*Class*	*School*	*ID*	*Class*	*School*
**Afternoon**	0.52	0.34	0.30	0.28	0.034	0.014
**Weekend**	^a^	0.41	0.31	^a^	0.09	0.06

^a^For weekday-weekend analyses, observations on the individual level were aggregated to relative frequency scores for the two respective periods and thus no ICC can be determined

In addition, we also freely estimated the slope parameters for each predictor on student (ID), class, and school levels and gradually reduced complexity in the model by removing those random effects on the class and school levels that were not credibly different from zero. The final models were chosen according to best model fit, using Markov Chain Monte Carlo (MCMC)-performance as an indicator. As all response variables are skewed, we fitted the data using a skew-normal distribution as a likelihood function, which captures the data better than the normal distribution.

The general model for the afternoon vs. morning analysis using brlm-syntax is as follows:

$$\mathrm{brm}(\mathrm{mvbind}\left({\mathrm{LPA}}_{\mathrm{afternoon}},{\mathrm{MVPA}}_{\mathrm{afternoon}}\right)\sim{\mathrm{ST}}_{\mathrm{morning}}+\mathrm{ses}+\mathrm{environment}+\mathrm{bmi}+\mathrm{age}+(1+{\mathrm{ST}}_{\mathrm{morning}}+\mathrm{ses}+\mathrm{environment}+\mathrm{bmi}+\mathrm{age}\left|\mathrm j\right|\mathrm{id})+\left(1+{\mathrm{ST}}_{\mathrm{morning}}+\left|\mathrm k\right|\mathrm{class}\right)+(1+{\mathrm{ST}}_{\mathrm{morning}}+\left|\mathrm l\right|\mathrm{school})$$

where LPA_afternoon as well as MVPA_afternoon are the dependent variables and everything on the right side of ∼ specifies predictors (ST_morning, SES, environment, weight status and age). For both the observation and group level terms the + is used to separate effects from each other. Group level terms are of a form (coefficient | group) where a coefficient contains one or more variables whose effects are assumed to vary with the levels of the grouping factor given in the group. In our sample, we differentiated between ID, class and school level. On a school and class level, only the association of LPA_afternoon/ MVPA_afternoon and ST_morning is analysed related to varying effects. Intercepts for the respective levels are denoted with the constant 1.

This term was adjusted for weekend analyses, containing LPA_weekend as well as MVPA_weekend as dependent variables and ST_weekday as one of the predictors. The rest is treated in the same way as in the first model, except on the ID-level, we simplified the formula by reducing the variables to ST_weekday:

$$\mathrm{brm}(\mathrm{mvbind}\left({\mathrm{LPA}}_{\mathrm{weekend}},{\mathrm{MVPA}}_{\mathrm{weekend}}\right)\sim{\mathrm{ST}}_{\mathrm{weekday}}+\mathrm{ses}+\mathrm{environment}+\mathrm{bmi}+\mathrm{age}+\left(1+{\mathrm{ST}}_{\mathrm{weekday}}\left|\mathrm p\right|\mathrm{id}\right)+\left(1+{\mathrm{ST}}_{\mathrm{weekday}}\left|\mathrm q\right|\mathrm{class}\right)+\left(1+{\mathrm{ST}}_{\mathrm{weekday}}\left|\mathrm r\right|\mathrm{school}\right)$$

To determine statistical significance, a 95% high density interval (HDI) was used for weekdays (morning-afternoon) to display posterior distributions. Due to the skewness of the response variables, the posterior distributions are not symmetrical and the HDI captures this better than equally-tailed intervals [65]. Yet, the HDI can be interpreted in the same way as equal-tailed summary credible intervals. MCMC simulation indicated a sufficient number of independent draws to warrant 95% credibility. For the weekday-weekend analysis, MCMC quality was sufficient to use the 90% HDI to indicate significant findings [65]. Additionally, the 60% HDI trends were calculated.

#### Statistical programs

 All statistical tests and illustrating graphs were performed using R version 4.1.1 [66] and Excel version Professional Plus 2016 [67]. Inferential models were fit using the R-package “brms” [68]. Summary statistics and highest density intervals have been calculated with “parameters” [69], and plots have been generated using “bayesplot” [70].

## Results

### Sociodemographic data

Valid accelerometer data from 370 girls with a mean age of 11.6 (*SD* = 0.5) years were available (Table 2). Regarding the weight status of the girls, the mean BMI value was 19.5 kg · m^−2^ (*SD* = 3.7). Related to the classification of Kromeyer-Hauschild, Wabitsch [71], which provides percentile curves of the BMI of a representative sample of children and adolescents in Germany, the BMI mean value of 19.5 kg · m^−2^ indicates the 75^th^ percentile in adolescent girls. With possible values between 16 and 90 [59], the score in our sample ranged between 16 and 89, and the mean SES in our study was 50.9 (*SD* = 16.0), indicating an overall medium SES with respect to the included girls. The built environmental factors of participants ranged from 0 to 10 with a mean value of 6.8 out of a possible 10.

**Table 2:** Descriptive data from included participants (Mean (SD))

Sample (*n* = 370)	Mean (SD)
***Sociodemographic data***	
Age (years)	11.6 (0.5)
Body mass index (kg · m^−2^)	19.5 (3.7)
Socioeconomic status	50.9 (16.0)
Environmental factor	6.8 (2.2)
***Physical activity on weekdays***	
ST in the morning (min/morning)	283.3 (54.9)
ST in the afternoon (min/afternoon)	255.3 (61.7)
LPA in the morning (min/morning)	101.5 (37.9)
LPA in the afternoon (min/afternoon)	85.5 (33.2)
MVPA in the morning (min/morning)	44.2 (16.9)
MVPA in the afternoon (min/afternoon)	42.7 (22.0)
***Physical activity on weekend days***	
ST (min/day)	430.4 (97.5)
LPA (min/day)	150.8 (55.1)
MVPA (min/day)	30.7 (11.6)

### Descriptive analysis of sedentary behaviour and physical activity

**Fig. 2:**
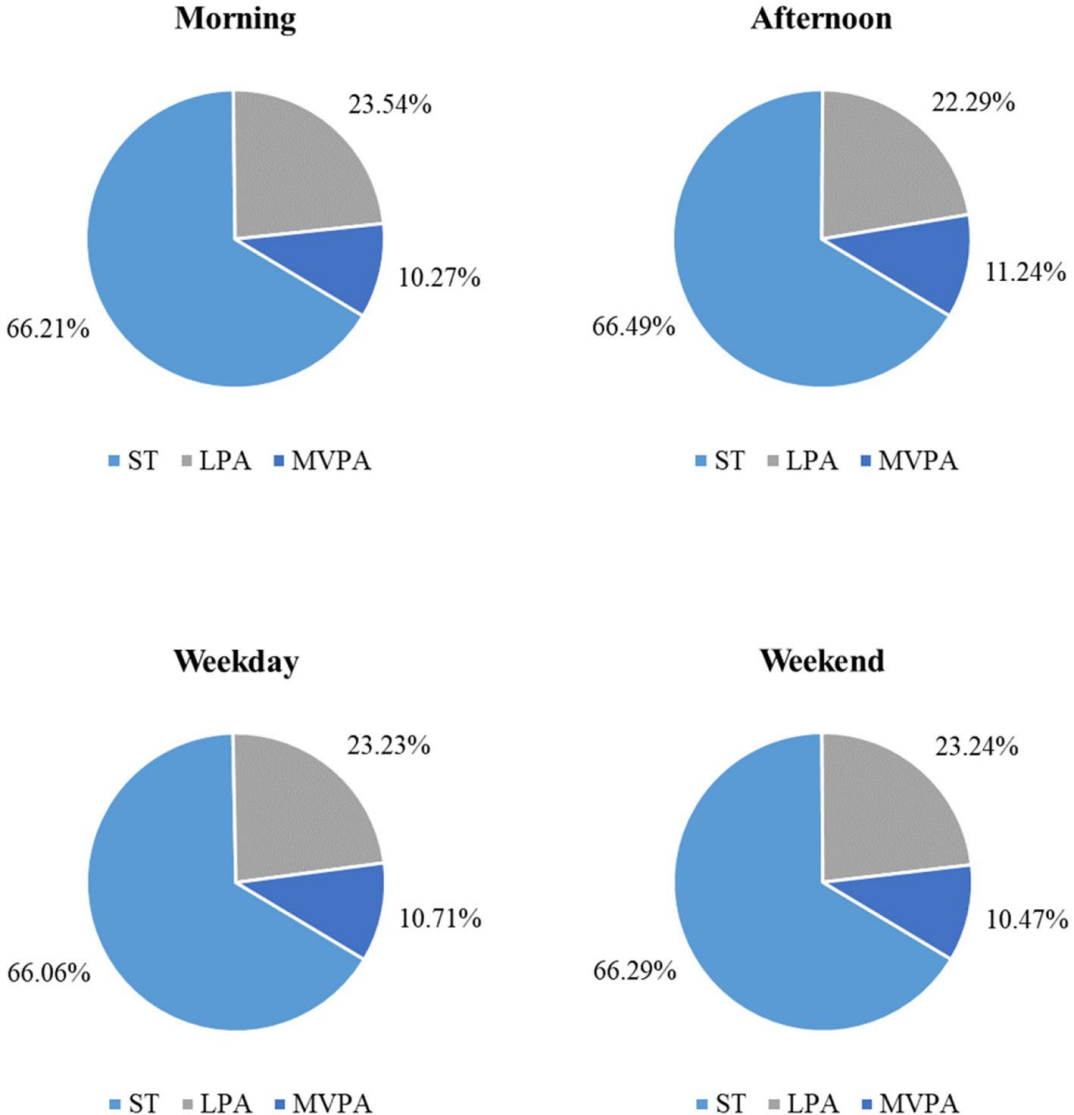
Relative time of sedentary behaviour and physical activity in the morning and afternoon and on weekdays and the weekend

PA and ST levels were calculated for weekdays as well as for weekend days (Table 2). We analysed relative PA and ST levels due to the various wear times of the accelerometer for girls (Fig. 2).

#### Morning-time vs. afternoon-time during the week

**Fig. 3:**
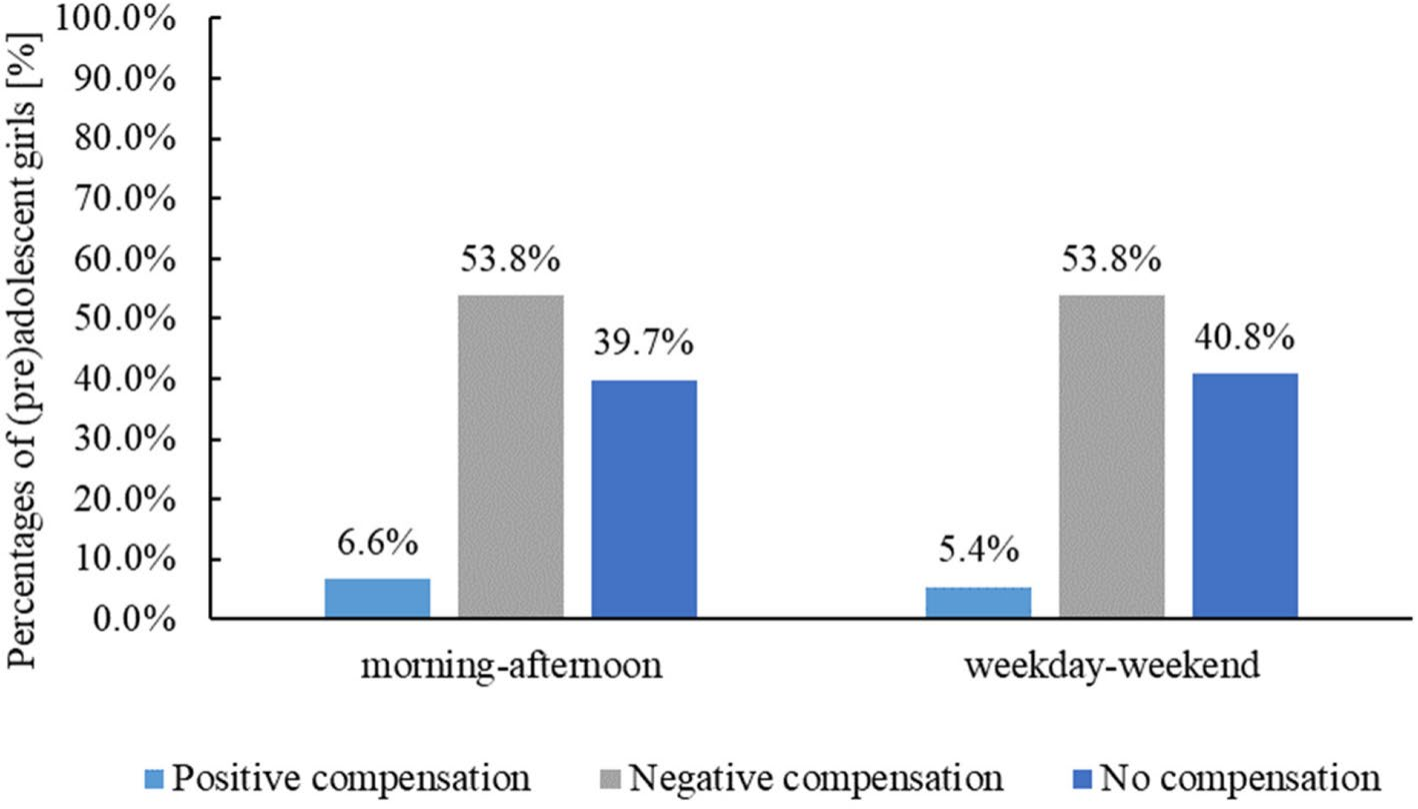
Negative, positive or no compensatory behaviour. Percentages of participants showing or not showing a compensatory behaviour at all observation points in the afternoon (left) and on the weekends (right)

During the mornings, the girls spent 66.21% per day being sedentary and in the afternoon the amount was similar at 66.49%. In the morning, LPA levels were slightly greater than in the afternoon (23.54% vs. 22.29%) whereas MVPA levels were slightly higher in the afternoon (10.27% vs. 11.24%, see Fig. 3).

#### Weekdays vs. weekends

During weekdays, 66.06% of the (pre)adolescents’ time was spent on ST and during the weekend this amount was similar with 66.29% spent being sedentary. Furthermore, relative values indicated similar LPA levels in (pre)adolescent girls between weekdays and weekend (23.23% vs. 23.24%) (see Fig. 2).

### Descriptive analysis of compensatory behaviour

To assess compensatory behaviour, descriptive analyses were conducted related to the morning and afternoon period as well as the weekend and weekdays (see Figs. 3).

#### Morning-time vs. afternoon-time during the week

**Fig. 4:**
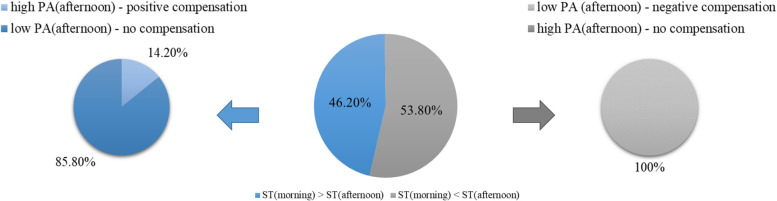
Differentiated analysis of compensatory behaviour. Percentages of observation points indicating a negative or no compensatory behaviour after having low ST levels in the morning (left) and percentages of observation points indicating a positive or no compensatory behaviour after having high ST levels in the morning (right)

Descriptive analyses indicated compensatory behaviour in 689 of 1,142 (60.4%) valid observation points during weekdays with 6.6% positive and 53.8% negative points of compensation (Fig. 3). Differentiated analysis indicated relatively more ST in morning compared to the afternoon in 528 observations, in which, as a consequence, allowed positive compensation to occur. In our sample, in 75 of these 528 observation points (14.2%) positive compensatory behaviour with more LPA and MVPA in the afternoons was seen. On the other hand, all observation points in girls with more PA in the morning, which is the basis for a negative compensation, showed a negative compensation of the activity with more ST in the afternoons (Fig. 4).

#### Weekday vs. weekend

**Fig. 5:**
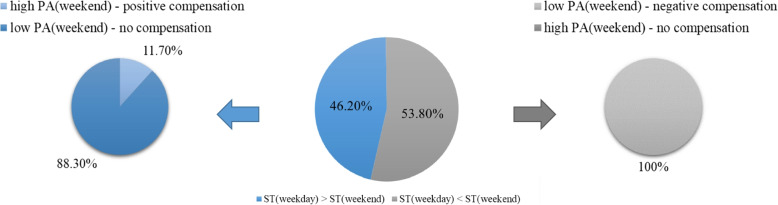
Differentiated analysis of compensatory behaviour. Percentages of observation points indicating a negative or no compensatory behaviour after having low ST levels during weekdays (left) and percentages of observation points indicating a positive or no compensatory behaviour after having high ST levels during the weekdays (right)

On weekend days, compensation was revealed in 219 of 370 girls (59.2%) with 5.4% positive and 53.8% negative compensatory behaviour (Fig. 3). Overall, 171 girls had more ST during weekdays and thus the potential to compensate positively, but only 11.7% (*N* = 20) actually displayed more LPA and MVPA on weekend days. On the other hand, all girls (*N* = 199) with higher PA levels during weekdays compared to weekend days, i.e., those who were particularly positioned to negatively compensate, demonstrated this behaviour. This means, that they compensated the higher PA-levels during the week with higher ST on weekend days (Fig. 5).

### Inferential analysis of physical activity

#### Morning-time vs. afternoon-time during the week

Inferential statistical analyses investigated if sedentary time in the mornings (ST1) would predict PA in the afternoons (LPA2, MVPA2), dependent on SES, age, and weight status (Table 3).

**Table 3:** Associations on an observational level between PA levels in the afternoon and ST in the morning and sociodemographic variables

Parameter	Median	60% HDI	95% HDI	Pd (%)	Rhat	ESS
*LPA2*						
Intercept	-0.01	[-0.08, 0.06]	[-0.19, 0.17]	55.3	1.000	18,490
**ST1**	**-0.44**	**[-0.49, -0.38]**	**[-0.56, -0.31]**	**100**	**1.000**	**17,555**
SES	-5.14e-03	[-0.04, 0.03]	[-0.09, 0.07]	54.81	1.000	17,784
**Weight status**	**-0.10**	**[-0.13, -0.07]**	**[-0.17, -0.02]**	**99.5**	**1.000**	**18,071**
**Age**	**-0.06**	**[-0.08, -0.02]**	**[-0.12, 0.02]**	**93.74**	**1.000**	**18,320**
*MVPA2*						
**Intercept**	**0.07**	**[0.02, 0.12]**	**[-0.04, 0.19]**	**88.59**	**1.000**	**17,800**
**ST1**	**-0.19**	**[-0.25, -0.14]**	**[-0.33, -0.06]**	**99.60**	**1.000**	**18,188**
SES	5.23e-03	[-0.03, 0.04]	[-0.07, 0.08]	55.14	1.000	18,117
weight status	-0.03	[-0.06, 0.01]	[-0.10, 0.05]	77.35	1.000	18,099
**age**	**-0.02**	**[-0.10, -0.04]**	**[-0.14, 0.00]**	**97.64**	**1.000**	**17,054**

Environment was included as a confounder

*Abbreviations: LPA2* LPA in the afternoon, *MVPA2* MVPA in the afternoon, *ST1* ST in the morning, *Pd (%)* Probability of direction of the sign ( ±), *Rhat* Scale Reduction Factor of the MCMC simulation, *ESS* Effective Sample Size of the MCMC simulation

Fixed effects for the LPA2-model showed a negative, medium to large association between ST in the morning and LPA in the afternoon (β = -0.44; 95% HDI = [-0.56, -0.31]) which indicated that girls with more ST in the morning showed lower LPA in the afternoon. Furthermore, a small negative association was estimated between weight status and LPA in the afternoon with a low effect size (β = -0.10; 95% HDI = [-0.13, -0.07]). SES was not associated with LPA behaviour in the afternoon in (pre)adolescent girls, however, a small negative association was seen between age and LPA in the afternoon (β = -0.06; 60% HDI = [-0.08, -0.02]). MVPA levels in the afternoon were also negatively associated with a medium effect size of ST in the morning (β = -0.19; 95% HDI = [-0.33, -0.06]). Moreover, a small negative association between MVPA in the afternoon and age (β = -0.02; 60% HDI = [-0.10, -0.04]) is shown in the 60% HDI.

Besides the fixed effects, we investigated associations on different group levels (Table 4). On the individual identification **(ID) level,** the intercept in the LPA2-model indicated significant differences between the individual girls in the afternoon (*SD* = 0.34, 95% HDI = [0.26, 0.42]). The direction of the association between ST in the morning time and LPA in the afternoon was not significantly different between the girls (*SD* = 0.09, 95% HDI = [0.00, 0.21]). Furthermore, the direction of the slopes of SES, weight status and age did not significantly vary between them.

**Table 4:** Associations on group level between PA levels in the afternoon and ST in the morning and sociodemographic variables

Parameter (SD's)	Median	60% HDI	95% HDI	Pd (%)	Rhat		ESS
*ID level*							
**LPA2_Intercept**	**0.34**	**[0.31, 0.38]**	**[0.26, 0.42]**	**100**	**1.000**		**15,511**
LPA2_ST1	0.09	[0.00, 0.11]	[0.00, 0.21]	100	1.000		11,894
LPA2_SES	0.05	[0.00, 0.06]	[0.00, 0.14]	100	1.000		14,819
LPA2_weight status	0.04	[0.00, 0.13]	[0.00, 0.13]	100	1.000		15,641
LPA2_age	0.06	[0.00, 0.07]	[0.00, 0.14]	100	1.000		15,869
**MVPA2_Intercept**	**0.37**	**[0.34, 0.41]**	**[0.29, 0.45]**	**100**		**1.000**	**16,618**
MVPA2_ST1	0.06	[0.00, 0.07]	[0.00, 0.17]	100		1.000	14,183
MVPA2_SES	0.06	[0.00, 0.07]	[0.00, 0.16]	100		1.001	13,883
MVPA2_weight status	0.05	[0.00, 0.07]	[0.00, 0.16]	100		1.000	15,555
MVPA2_age	0.04	[0.00, 0.05]	[0.00, 0.12]	100		1.000	16,969
*Class Level*							
LPA2_Intercept	0.08	[0.00, 0.10]	[0.00, 0.22]	100		1.000	15,375
**LPA2_ST1**	**0.10**	**[0.03, 0.14]**	**[0.00, 0.22]**	**100**		**1.000**	**17,143**
MVPA2_Intercept	0.07	[0.00, 0.08]	[0.00, 0.18]	100		1.000	17,553
MVPA2_ST1	0.06	[0.00, 0.07]	[0.00, 0.18]	100		1.000	17,348
*School level*							
**LPA2_Intercept**	**0.25**	**[0.17, 0.32]**	**[0.09, 0.47]**	**100**		**1.000**	**16,303**
LPA2_ST1	0.10	[0.00, 0.13]	[0.00, 0.25]	100		1.000	16,917
MVPA2_Intercept	0.07	[0.00, 0.09]	[0.00, 0.21]	100		1.000	17,137
**MVPA2_ST1**	**0.15**	**[0.08, 0.23]**	**[0.00, 0.32]**	**100**		**1.000**	**16,984**

Environment was included as a confounder

*Abbreviations: LPA2* LPA in the afternoon, *MVPA2* MVPA in the afternoon, *ST1* ST in the morning, *Pd (%)* Probability of direction of the sign ( ±), *Rhat* Scale Reduction Factor of the MCMC simulation, *ESS* Effective Sample Size of the MCMC simulation

Regarding MVPA levels in the afternoon, a significant difference between the girls on the ID level (*SD* = 0.37, 95% HDI = [0.29, 0.45]) was indicated by the large standard deviation of the intercept (MVPA2_Intercept). No differences between the girls concerning the association between ST in the morning and MVPA levels in the afternoon were indicated (*SD* = 0.06, 95% HDI = [0.00, 0.17]).

Regarding differences on the **class cluster-level** related to LPA and MVPA behaviour in the afternoon, there was only a small trend of significant differences in the association between ST in the morning and LPA in the afternoon (*SD* = 0.10, 60% HDI = [0.03, 0.14]).

However, on the **school cluster-level**, girls differed in their LPA in the afternoon (*SD* = 0.25, 95% HDI = [0.07, 0.55]). Related to the association between ST in the morning and MVPA behaviour in the afternoon, the 60% HDI ([0.09, 0.47]) indicated a trend of difference between the girls according to school level.

#### Weekday vs. weekend

The associations between ST on weekdays and PA on weekends were also assessed.

The model revealed a medium to large sized negative association between ST on weekdays and LPA (β = -0.49; 90% HDI = [-0.60, -0.52]) as well as MVPA levels (β = -0.39; 90% HDI = [-0.53, -0.12]) on weekend days. Additionally, some trends can be seen: for example, weight status and age revealed negative associations with LPA on weekend days (β = -0.09; β = -0.02). Moreover, for MVPA levels on weekend days, SES tended to have a positive association (β = 0.04; 60% HDI = [0.01, 0.08]) (see Table 5).

**Table 5:** Associations on an observation level between PA levels on weekends and ST on weekdays and sociodemographic variables

Parameter	Median	60% HDI	90% HDI	Pd (%)	Rhat	ESS
*LPAWknd*						
Intercept	-0.05	[-0.11, 0.01]	[-0.17, 0.08]	74.67	1.000	7652
**STWkdy**	**-0.49**	**[-0.54, -0.41]**	**[-0.60, -0.52]**	**100**	**1.000**	**6149**
SES	4.99e-3	[-0.04, 0.03]	[-0.07, 0.06]	56.11	1.001	5713
**Weight status**	**-0.09**	**[-0.10, -0.03]**	**[-0.13, 0.01]**	**93.71**	**1.000**	**4398**
**Age**	**-0.02**	**[-0.08, -0.01]**	**[-0.11, 0.03]**	**87.04**	**1.000**	**4657**
*MVPAWknd*						
Intercept	0.07	[-0.03, 0.16]	[-0.12, 0.26]	73.22	1.000	9415
**STWkdy**	**-0.39**	**[-0.46, -0.31]**	**[-0.53, -0.24]**	**99.99**	**1.000**	**9726**
SES	0.04	[0.01, 0.08]	[-0.03, 0.12]	84.44	1.000	9655
Weight status	-0.02	[-0.06, 0.02]	[-0.09, 0.06]	64.94	1.000	8610
Age	-0.02	[-0.05, 0.02]	[-0.09, 0.05]	65.28	1.000	8660

Environment was included as a confounder

*Abbreviations: LPAWknd* LPA on weekend, *MVPAWknd* MVPA on weekend, *STWkdy* ST on weekday, *Pd (%)* Probability of direction of the sign ( ±), *Rhat* Scale Reduction Factor of the MCMC simulation, *ESS* Effective Sample Size of the MCMC simulation

Besides the fixed effects for all observations of LPA and MVPA on weekends, random effects on the ID, school and class levels were also investigated (Table 6).

**Table 6:** Associations on group level between PA levels on weekends and ST on weekdays and sociodemographic variables

Parameter (SD's)	Median	60% HDI	90% HDI	Pd (%)	Rhat	ESS
*ID level*						
**LPAWknd_Intercept**	**0.56**	**[0.50, 0.63]**	**[0.43, 0.68]**	**100**	**1.012**	**230**
**LPAWknd_STWkdy**	**0.31**	**[0.24, 0.40]**	**[0.12, 0.46]**	**100**	**1.003**	**1187**
**MVPAWknd_intercept**	**0.38**	**[0.32, 0.44]**	**[0.26, 0.49]**	**100**	**1.000**	**2824**
MVPAWknd_STWkdy	0.12	[0.00, 0.14]	[0.00, 0.24]	100	1.000	10,181
*Class level*						
**LPAWknd_Intercept**	**0.19**	**[0.12, 0.26]**	**[0.05, 0.33]**	**100**	**1.000**	**3607**
**LPAWknd_STWkdy**	**0.12**	**[0.13, 0.24]**	**[0.06, 0.31]**	**100**	**1.000**	**5254**
**MVPAWknd_Intercept**	**0.20**	**[0.12, 0.26]**	**[0.04, 0.34]**	**100**	**1.000**	**6120**
**MVPAWknd_STWkdy**	**0.14**	**[0.07, 0.20]**	**[0.00, 0.25]**	**100**	**1.000**	**6132**
*School level*						
LPAWknd_Intercept	0.14	[0.04, 0.20]	[0.00, 0.28]	100	1.000	3901
LPAWknd_STWkdy	0.09	[0.00, 0.11]	[0.00, 0.21]	100	1.000	4243
**MVPAWknd_Intercept**	**0.33**	**[0.22, 0.41]**	**[0.13, 0.55]**	**100**	**1.000**	**7778**
**MVPAWknd_ST1**	**0.20**	**[0.12, 0.29]**	**[0.01, 0.36]**	**100**	**1.000**	**6822**

Environment was included as a confounder

*Abbreviations: LPAWknd* LPA on weekend, *MVPAWknd* MVPA on weekend, *STWkdy* ST on weekday, *Pd (%)* Probability of direction of the sign ( ±), *Rhat* Scale Reduction Factor of the MCMC simulation, *ESS* Effective Sample Size of the MCMC simulation

On the **ID level**, LPA as well as MVPA on weekend days differed significantly between the girls (LPA *SD* = 0.56; 90% HDI = [0.43, 0.68], MVPA *SD* = 0.38; 90% HDI = [0.26, 0.49]), as did the direction of slope-parameters of ST on weekdays on LPA on weekend days (*SD* = 0.31; 90% HDI = [0.12, 0.46]).

This can also be seen on the **class cluster-level.** Additionally, the ST on weekdays slope concerning weekends' MVPA tended to vary across the girls (*SD* = 0.14; 60% HDI = [0.07, 0.20]).

**On the school cluster level,** the standard deviation of the slope parameters indicated significant differences in MVPA levels on weekend days between the girls at different schools (*SD* = 0.33; 90% HDI = [0.13, 0.55]). Differences in LPA levels on weekends were only significant at 60% HDI (*SD* = 0.14; 60% HDI = [0.04, 0.20]), thus, indicating a trend. Regarding the slope of ST on weekdays concerning MVPA on weekend days, a variation between the girls on a school level can be seen (*SD* = 0.20; 90% HDI = [0.01, 0.36]).

## Discussion

The aim of the present study was to investigate, with respect to (pre)adolescent girls in Germany, whether ST in the morning is compensated by PA in the afternoon and, whether ST on weekdays is compensated by PA behaviour on weekends. Additionally, we examined whether these relationships are moderated by the SES, age or weight status of the (pre)adolescent girls.

By applying a within-person design, compensation analysis related to afternoon and weekend PA was conducted for every single girl in our study. Our findings reveal compensatory behaviour in the afternoon period in 60.4% of the observation points across all 370 girls who participated in this study. For a better understanding, we would like to review the meaning of observation points ones more. These refer to the fact that one girl can produce values on several days in the morning and in the afternoon over a period of seven days. Thus, girls could have multiple observation points. Related to weekend PA, 59.2% of the (pre)adolescent girls indicated compensatory behaviour. We conducted differentiated analyses and found in our sample that during weekdays 614 observation points in girls indicated lower ST in the morning compared to afternoon. All these showed a negative compensation with lower PA in the afternoon. Similar results can be seen regarding weekend compensation: all girls (*N* = 199) with lower ST levels during the week compared to the weekend showed lower PA levels on the weekend. Thus, all these girls compensated negatively. Nevertheless, our data indicate that more ST in the morning or on weekdays is compensated positively by more PA in the afternoon or on weekends, even if the number of girls doing so in the present sample was small. In the afternoon, only 14.2% (75 of 528) of the observation points with more ST levels in the morning showed an increase of PA levels in the afternoon, and on weekends less than 12% compensated positively for more ST during the week with more PA on the weekend (20 of 171 girls). Interestingly, the number of girls who did not compensate, is represented only by those girls who had high ST levels in the morning/on weekdays and did not increase their PA levels in the afternoons/on weekends. Thus, they accumulate throughout the day and entire week low PA levels. This result can also be seen regarding the negative association between ST levels and PA levels (see Tables 3 and 5).

The latest reviews summarising existing literature related to compensatory behaviour in children and adolescents reveal similar results [38, 39]. In the present study, in the afternoons as well as on weekends, 53.8% of observation points indicated negative compensatory behaviour and our review [38] also indicates negative compensatory behaviour in half of the studies that focused on daily PA behaviour [40–46]. The latest review by Swelam, Verswijveren [39] analysing youths and adults compensatory behaviour indicates compensatory behaviour in one-third of the 5 to 18-years-olds, thereby confirming the inconsistent results of the present study.

In addition to the negative compensatory behaviour in the present sample, our data indicate positive compensatory behaviour in a few girls. Until now, only two studies revealed positive compensatory behaviour in children and adolescents: Tudor-Locke, Lee [29] reported a positive compensation in girls with lower PA levels in school and increased PA levels out of school. Thus, total PA related to the entire day did not differ between girls with higher and lower in school PA in this study. Another study on German children indicated higher MVPA levels than usual the day after higher than usual ST levels [48]. Interestingly, positive compensation in differentiated analyses was seen more frequently in the afternoon compared to weekend days (14.2% vs. 11.7%). This could be explained by generally higher PA levels during the week compared to weekends [40, 72, 73]. Furthermore, literature suggests that weekends were not used for PA by children and adolescents [73], even if there was enough time, because children and adolescents did not go to school. In addition, training times for sports clubs more frequently occur during the week in the afternoon/evening when schools offer opportunities to be active (e.g., active commuting to school, extracurricular PA, PE) [74–76].

In this context, we would like to highlight the differentiation of positive and negative compensation in our study, as we are the first to do so. Due to our descriptive analysis of compensation we were able to determine for each girl/observation point the individual PA behaviour. This makes it possible to determine and differentiate between positive or negative compensatory behaviour in girls, or the absence thereof. Most of the existing studies analysed compensation across the mean values of the group and thus only obtained one value for each timespan [36, 77–80]. Nigg, Burchartz [48] also take the individual mean values of every individual into account for compensation analysis; nevertheless, the result of the multilevel analysis indicated one mean value under consideration for all the participants’ individual means.

Furthermore, related to our additional multilevel analysis, compensatory behaviour differs between the girls. Nevertheless, these differences in the compensatory behaviour were not related to any of the sociodemographic variables we included, such as SES, age or weight status. Thus, we assume that it depends on individual or social factors (e.g. intrinsic motivation as well as social support). Furthermore, Swelam, Verswijveren [39] also stated individual reasons for compensation like fatigue, time constraints, lack of motivation, drive to be inactive, fear of overexertion, and autonomous motivation. These results are confirmed by the Eurobarometer Study [81], which investigated, among other things, the barriers for engaging in PA. It was found that especially for younger adolescent girls barriers to PA are a lack of time followed by a lack of motivation and interest [81].

As we conclude, to avoid a negative compensation following more PA in the morning/on weekdays, it is important to support (pre)adolescents in their active lifestyle after school and especially on weekends. Therefore, it seems important to develop strategies to promote PA levels after school and on weekends, and these should also focus on changing the attitude and mind of the adolescents related to PA [26, 82, 83].

The importance of the schools as a promoter of PA is also confirmed when regarding our results on the class and school levels. Especially on a class level, differences can be seen between the girls regarding the association between ST in the morning/on weekdays and LPA in the afternoon/on weekend days. This means, that compensation occurs depending on class affiliation. MVPA levels and compensatory behaviour with MVPA were observed with regard to school level. Unfortunately, we do not have further information about the school profiles or timetables of the classes, which makes it difficult to interpret these findings. However, these factors seem to be relevant moderators of compensatory behaviour. Further studies should focus on differences between class and school levels by taking school profiles and timetables into account.

Lastly, we would like to focus on the timeframe of compensatory behaviour. Existing reviews indicate inconsistent results related to the timeframe of compensation. Overall, compensatory behaviour occurs within days, between days as well as over (several) weeks [27, 38, 39]. Gomersall, Rowlands [27] hypothesised that the timeframe for compensation is unlikely to be day to day. However, our review revealed that most included studies investigating PA behaviour within days showed that half indicated compensation [38] as it can also be seen in the current study. On the other hand, the review of Swelam, Verswijveren [39] found as much compensatory behaviour within as between days (approx. 25%). Nevertheless, compensatory behaviour between weekdays and weekends was scarcely investigated [84]. Thus, our data expanded the current state of research related to weekend compensation and demonstrated compensatory behaviour in more than half of the girls (53.8%).

In summary, our findings reveal that half of the female sixth graders in our study showed a positive or negative compensatory behaviour. Thus, we can neither confirm nor reject the ActivityStat Hypothesis, stating that an increase of PA in one timespan is compensated by a decrease of PA in another timespan [27]. Overall, the results indicate that PA behaviour in the afternoon or on the weekends are predominantly individual and differ greatly between the girls. In addition, PA behaviour in our study was not associated with age, weight status or SES. Nevertheless, further variables regarding SES like possessing a car and/or bicycle should be considered and could be associated with compensatory behaviour. In addition, further compensation studies should focus on individual variables like social support as the literature has suggested differences in PA levels depending on social support [85]. Furthermore, intervention or health programmes targeting on a positive attitude towards PA should be implemented to highlight the importance of sufficient PA for health [26, 82].

### Strengths and limitations

The major strength of this study is its detailed insight into device-based assessed PA and ST behaviour during weekdays (morning, afternoon) as well as on weekends concerning (pre)adolescent girls, a high-risk population. This allows for conclusions about compensatory behaviour and potential moderators. This study expands the current research of the PA and ST of students due to its detailed analysis of a large sample size, which establishes a foundation for further investigations. Furthermore, we investigated and reported the compensatory behaviour of each individual and were thus able to differentiate between positive, negative and no compensation within the sample. Existing studies have mainly analysed compensation with mean PA values and this study is the first to investigate the compensatory behaviour of girls on different levels (ID, school, and class) and under the consideration of different moderators (age, weight status, SES).

Nevertheless, this study has several limitations. Further factors like seasonal and meteorological variability mediating PA and ST levels were not considered in the analysis. Furthermore, we did not analyse different segments during the school day to acquire more information about PA behaviour during school hours as applied to other studies [86]. Lastly, we do not have any information about the school profile and/or the timetables of the students which makes it difficult to interpret our findings related to school and class level effects. In this context, it would generally be helpful to give participants, in addition to the accelerometers, a diary in order to gain more information about their behaviour. Besides the school profile, the school district SES could be included in further studies, as an existing study revealed an association between the socioeconomic circumstances of the area the children lived in and health development [87].

## Conclusion

The present study investigated compensatory behaviour and moderating variables in a sample of sixth-grade girls (*N* = 370). Our results indicated compensatory behaviour in about 60% of all observation points, both in the afternoon and, on weekends. However, concerning this behaviour, only a few girls revealed a positive compensation by increasing their PA behaviour after a high amount of ST. On the other hand, all girls with low ST levels in the morning or on weekdays, compensated for this behaviour with lower PA levels in the afternoon or on weekend days. This behaviour directly prompts for action to maintain a high PA level throughout the entire day/week. Inferential analysis confirmed our results and implicated that there were intra-individual differences in the occurrence of a compensatory behaviour. However, relevant moderators explaining intra-individual differences were not identified. In the future, to prevent negative compensation, school-based interventions that have the potential to provide opportunities to be active, should not neglect (pre)adolescents’ out of school behaviour. Theoretical models [88] and empirical findings [89] indicate that PA promotion programmes are more effective if they address more than one level of influence (e.g., school and family). Furthermore, one important point could be to highlight the importance of PA for health and motivate them to stay active throughout the whole day.

## Data Availability

The datasets used and/or analysed during the current study are available from the corresponding author on reasonable request.

## Abbreviations

BMI: Body Mass Index
CPB: Compensatory physical behaviour
Cpm: Counts per minute
EE: Energy expenditure
ESS: Effective Sample Size of the MCMC simulation
HDI: High density interval
HISEI: Higher International Socioeconomic Index of occupational status
ICC: Intra-class correlation coefficient
ISEI: International Socioeconomic Index of occupational status
LPA: Light physical activity
MCMC: Markov chain Monte Carlo simulation
MVPA: Moderate-to-vigorous physical activity
PA: Physical activity
Pd (%): Probability of direction of the sign ( ±)
PE: Physical Education
Rhat: Scale Reduction Factor for MCMC simulation
SES: Socioeconomic status
ST: Sedentary time
WHO: World Health Organization
